# Does Continuous Bioaugmentation of Aerated Stabilization Basins Improve Performance? A Field Scale Trial With a Control

**DOI:** 10.1002/wer.70202

**Published:** 2025-11-12

**Authors:** Amanda Johansen Mattingly, Philip Pagoria, James Palumbo, Francis L. de los Reyes

**Affiliations:** ^1^ Department of Civil, Construction, and Environmental Engineering North Carolina State University Raleigh North Carolina USA; ^2^ National Council for Air and Stream Improvement Inc Cary North Carolina USA

**Keywords:** aerated stabilization basins, bioaugmentation, forest products, lagoons, pulp and paper, wastewater treatment

## Abstract

Continuous bioaugmentation is widely employed across the pulp and paper industry in attempts to improve the resilience of wastewater treatment systems or the performance of undersized (in terms of volume, aeration, or nutrient supply) systems. Bench and field scale research into bioaugmentation has shown that success is often unpredictable. A field scale trial at an aerated lagoon system treating pulp and paper mill wastewater was completed over a 6‐month period. The system consisted of two nearly identical trains of aerated stabilization basins (ASBs), one operated as a control and the other treated with a commercially available bioaugmentation product. The control and treated basins were then switched to minimize train‐specific effects. Throughout the trial, changes in soluble or total biochemical oxygen demand (sBOD5 and TBOD5, respectively) or total suspended solids (TSS) at the first of the two ponds in series were not associated with bioaugmentation. In the second set of ponds, bioaugmentation was associated with 6.0 ± 2.6 mg/L higher TBOD5 and 12.4 ± 5.2 mg/L higher TSS. Further, 16S rRNA gene sequencing identified high levels of *Thiothrix* in the bioaugmented train, whereas TSS data between the trains diverged. This provided evidence that the significant difference in BOD and TSS was likely due to a microbial community dominated by a filamentous bacterial bloom rather than bioaugmentation.

## Summary

- Bioaugmentation studies, with controls, at full scale wastewater systems are rare.
- Full‐scale tests with control reactors at a pulp and paper facility were performed.
- Comprehensive physical, chemical, and microbial parameters were measured.
- No significant improvement in general performance observed due to bioaugmentation.
- Bioaugmentation was not associated with a change in the microbial community.

## Graphical abstract

Full‐scale bioaugmentation tests, with controls, in aerated stabilization basins treating pulp and paper mill wastewater were performed. The results from physical, chemical, and DNA sequencing analyses showed that bioaugmentation was not associated with a significant change in microbial communities nor significant improvement in general performance.

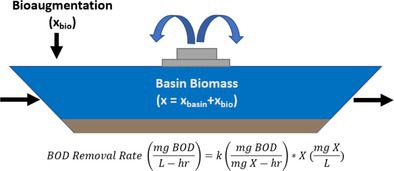

## 1 Introduction

Bioaugmentation has long been a strategy employed by pulp and paper biological wastewater treatment systems to attempt to improve performance in the face of capacity issues or periods of wastewater treatment plant stress. Bioaugmentation, the practice of adding an externally cultured biomass (either a selected bacterial strain or mixed culture) to a wastewater treatment process to improve performance, is often of the general type (for improvement in BOD and TSS) in the pulp and paper industry but is used in targeted applications as well. Targeted applications are those where a particular strain capable of performing a unique function is added (e.g., the addition of resin acid‐degrading bacteria to a system undergoing a sudden load of resin acid‐containing material) (Yu and Mohn 2002). Both general and targeted bioaugmentation processes can be accomplished by adding a preadapted pure strain, a preadapted microbial consortium, genetically engineered microorganisms, or the relevant genes to the target biodegradation process such that those genes may be transferred by conjugation into microorganisms in the system (Herrero and Stuckey 2015).

In theory, inadequate performance in an aeration basin may be due to an insufficient number of microorganisms in the basin, and bioaugmentation could increase the amount of biomass in the system capable of degrading the contaminant of interest. The addition of a relevant strain or consortia of microorganisms could change or reinforce the targeted microbial function. Bioaugmentation can thus be thought of as microbial immigration, and research has shown that immigrating microbial communities can play a large role in defining the microbial community structure of a bioreactor (Nemergut et al. 2013; Wells et al. 2014; Vellend 2010; Smith et al. 2024). Thus, for a bioaugmentation product to work, the introduced strains must grow and reproduce in the treated reactor, which can be limited by competition with indigenous microorganisms, predation, presence of bacteriophages, or failure to acclimate to environmental conditions (Herrero and Stuckey 2015).

At typical dosage rates in the pulp and paper industry, there is a concern that the manufacturer's recommended dosages are insufficient to affect the composition of the microbial community (Mattingly 2021). The overwhelming majority of studies in the peer‐reviewed literature are at the bench scale and assess targeted approaches (Mattingly 2021). Though many demonstrated improved performance over a control, studies at full scale have demonstrated little to no benefit and often are complicated by poor controls (inadequate characterization before and after treatment) or lack of statistical analysis (Mattingly 2021). Given the difficulties in translating bioaugmentation at the bench scale to full scale implementation, there is a need for independent full‐scale studies that pair wastewater treatment performance data with microbial community analysis. In this work, a typical generalized bioaugmentation installation was evaluated at a full‐scale pulp and paper aerated stabilization basin (ASB) system, with a full‐scale control reactor side by side with the treatment reactor. After one experimental run and after a period of hydraulic turnover, the control and treatment reactors were switched to minimize treatment train effects. The objectives of this study were to assess if the addition of a commercially available general bioaugmentation product as typically applied by the pulp and paper industry to ASBs could:

1. Improve performance of the aerated lagoon as measured by BOD and TSS.
2. Affect the composition of the microbial community present in the aerated lagoon.

This study represents one of the few full‐scale, controlled experiments of continuous general bioaugmentation with statistical and microbial community analyses.

## 2 Materials and Methods

### 2.1 Experimental Design

Two aerated stabilization basin (ASB) trains were operated with and without addition of the bioaugmentation product. The field scale trial was operated in two phases (Table 1). In Phase 1, Train 1 was operated with bioaugmentation, and Train 2 was operated as a control (without bioaugmentation). In Phase 2, the dosing was switched: Train 2 was operated with bioaugmentation, and Train 1 was operated as a control. At the beginning of each phase, there was a 2‐week period of dosing or stoppage of dosing prior to data collection for both trains. This allowed for three hydraulic retention times to ensure adequate hydraulic turnover between phases.

**TABLE 1:** Experimental matrix.

Time period	Train 1 bioaugmentation dose, kg/day (lb/d)	Train 1 bioaugmentation dose/avg. inf. BOD, kg/10,000 kg	Train 2 bioaugmentation dose, kg/day (lb/d)	Train 2 bioaugmentation dose/avg. inf. BOD, kg/10,000 kg
Phase 1 (March 30, 2020, to May 25, 2020)	4.5 (10)	2.5	0 (control)	0 (control)
Phase 2 (June 8, 2020, to August 5, 2020)	0 (control)	0 (control)	4.5 (10)	3.1

Dosage information for this product was gathered from a sample of seven facilities using the product, ranging from 0.2 to 4.3 kg product/10,000 kg influent BOD, with a median of 1 kg product/10,000 kg influent BOD (Table S1) (Mattingly 2021). A dose of 4.54 kg/day (2.5–3.1 kg product/10,000 kg influent BOD) of product was selected for this study, representing dosage that was two to three times the median value of the dosage data set.

### 2.2 Characteristics of Full‐Scale ASB Trains

**FIGURE 1:**
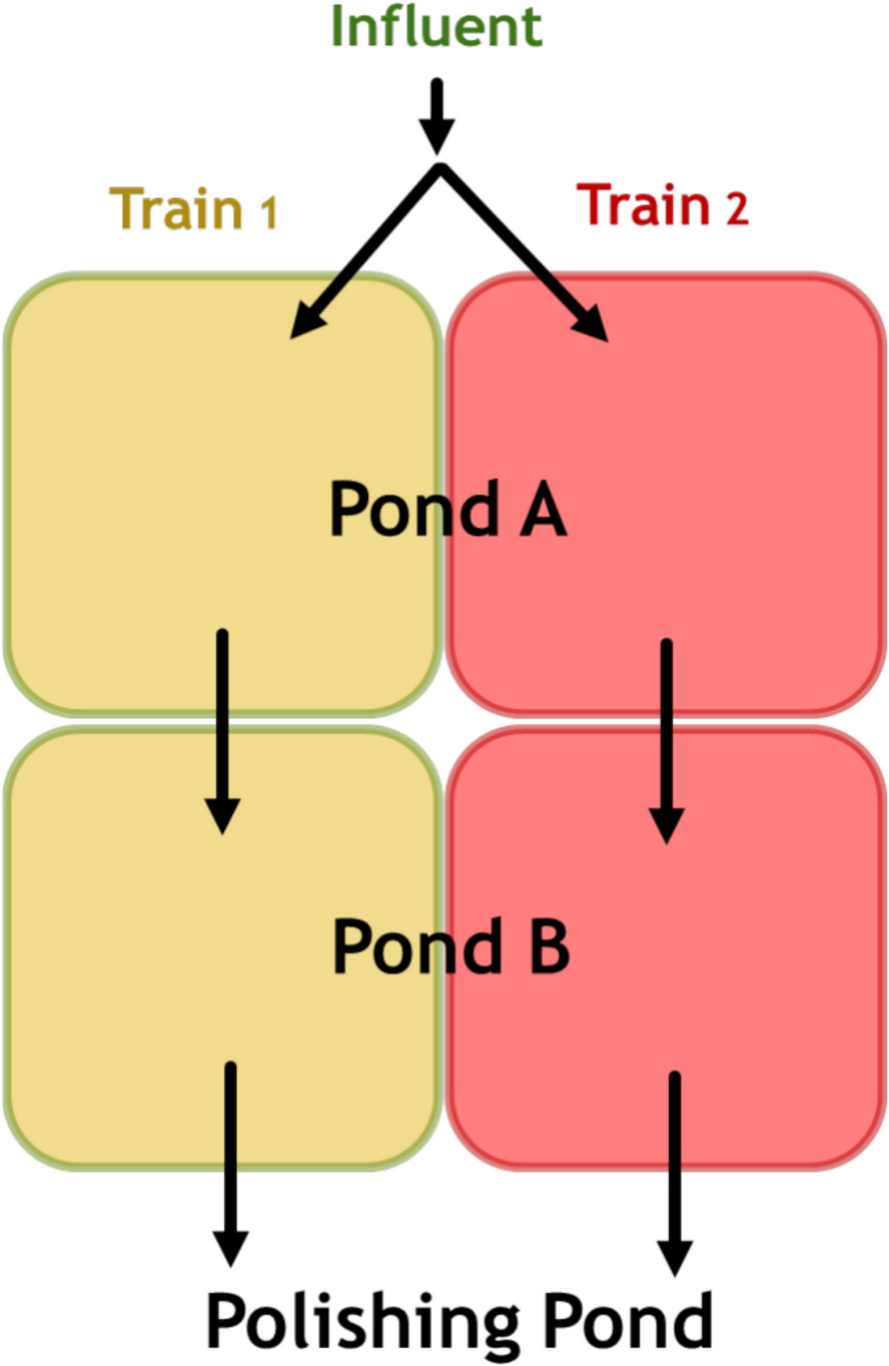
Process flow diagram of full‐scale aerated stabilization basins. At any given time, one train was bioaugmented, and the other one served as control; these were later switched to avoid train‐specific effects.

Wastewater treatment at the pulp and paper mill consisted of primary clarification and secondary treatment in aerated stabilization basins (ASBs), followed by a polishing pond (Figure 1). Wastewater that enters the ASBs from the primary clarifier first enters an influent splitter box, which divides the flow approximately in half between Trains 1 and 2. Each ASB train consists of two ponds (Ponds A and B), each with an HRT of ~2–3 days of hydraulic retention time (HRT). Tracer studies were performed to assess differences in HRT. Both trains had an estimated total retention time of 6.2 days, whereas Pond A had HRTs of 2.6 and 2.8 days for Trains 1 and 2, respectively. Each pond contains nine 75 HP surface aerators. The field scale trial was conducted on the two ASB trains only (the polishing pond was not included).

### 2.3 Bioaugmentation Incubator Setup, Seeding, Environment, and Performance Monitoring

The incubator system consisted of a main incubator tank with a liquid volume of approximately 992 L, a 1041‐L influent holding tank, a submersible influent feed pump, a positive displacement incubator feed pump, six air pumps (33.6 L/min at standard temperature) each equipped with two coarse diffused aeration stones, a recirculation pump, and overflow tubing that directed incubator outflow to either ASB Train A or B (Figure S1). The incubator feed pump operated for 13 min every hour at 3200 mL/min to provide raw influent wastewater to the incubator. The recirculation pump operated for 10 min every hour at a rate of 9800 mL/min to provide mixing. The air pumps operated continuously to maintain a DO concentration greater than 0.5 mg/L. The bioaugmentation product dosed was a commercially available dry product consisting of bacterial spores and growth media/bran carrier. Manual operation included feeding 4.54 kg of bioaugmentation product through the top of the incubator daily, as well as purging accumulated solids from the bottom of the unit. The system was operated at a 24‐h hydraulic retention time (HRT) per product specifications (feeding the entire contents to Pond A over 24 h). The selected HRT was checked with a bench scale experiment that verified bacteria were reproducing and no longer in a spore phase after a 24‐h incubation (Mattingly 2021). Initially, the bioaugmentation unit did not include a feed tank for the wastewater coming from the mill. Increasing temperatures as warmer weather set in prompted the use of an influent feed tank (1041 L), operated at a 24‐h HRT, to allow some time for the raw influent wastewater to decrease in temperature prior to dosing to the incubator. The bioaugmentation incubator was initially purged of solids twice per week. As warmer weather set in, the pH in the incubator began to drop, and daily purging was adopted to reduce fermentative activity affecting the pH in the incubator.

### 2.4 Analytical Methods

Biochemical oxygen demand, settleable solids, and total and volatile suspended solids (TSS and VSS) were measured according to Standard Methods (Rice et al. 2017). Chemical oxygen demand (COD) was analyzed using HACH High Range Plus COD vials (HACH, Loveland, CO) according to the manufacturer's instructions. Ammonia (NH3‐N) was analyzed via EPA Method 350.1. Filtered total phosphorus (FTP) was analyzed using a 0.45‐μm membrane filter and EPA Method 365.3.

Oxygen uptake rate was performed immediately upon sample collection at the sampling location according to Standard Methods 2710B (Rice et al. 2017). Cellular adenosine triphosphate (cATP) was analyzed using the Luminultra Technologies Ltd. (Fredericton, New Brunswick) QuenchGone21 Wastewater kits according to the manufacturer's instructions. Dissolved oxygen (DO), temperature, pH, and oxidation–reduction potential (ORP) were measured in the field using portable meters. Conductivity was measured in the lab according to Standard Methods 2510B (Rice et al. 2017).

Whole effluent toxicity tests were performed and data analyzed according to published methods (USEPA 2002). The test organism used in the analyses was *Ceriodaphnia dubia*. The reproduction IC25 value was reported for each of the tests. Heterotrophic plate counts (HPC) were analyzed in triplicate per Standard Methods 9215, using the spread plate method and R2A agar (Rice et al. 2017). Live samples were also analyzed via phase‐contrast microscopy. Samples were also dried onto slides and stained using Gram staining and Neisser staining methods (Jenkins et al. 2004). Three additional Gram stain slides were prepared and photographed (five locations per slide) in monochrome for analysis of particle size distribution. ImageJ Fiji software was used to analyze the photographs and collect particle size data, using manual thresholding (Schindelin et al. 2012). Particle size data from each slide were used to calculate the D43, as described in the equation,

$$D43=\frac{\sum_{i=1}^{j}\left({d_{i}}^{4}*v_{i}\right)}{\sum_{i=1}^{j}\left({d_{i}}^{3}*v_{i}\right)},$$

where *d*_*i*_ is the geometric mean of bin *i* and *v*_*i*_ is the fraction of particles in bin *i* over the total number of particles in the sample.

### 2.5 Statistical Analyses

To determine the effect of bioaugmentation on ASB performance (response variables), multiple linear regression models were considered. These models take the form:

$$Y_{i}=\beta_{0}+\sum_{j=1}^{k}\beta_{j}x_{\mathrm{ij}}+\varepsilon_{i},$$

where *Y* is the response variable, *i* is the index for the days in which the measurements were made, *β* denotes the effect for each predictor variable, *x*_*j*_ are the predictor variables, *j* is an index for the predictor variables included in the model, and *ε*_*i*_ denotes experimental error or variability not explained by the predictor variables. Experimental errors were assumed to be independent, normally distributed with constant variance. Because the ponds each had an HRT of 2–3 days, there was a concern that measurements close in time may be correlated (autocorrelation of the residuals). For Ponds A and B, models were fitted using 7‐day averages of the data to reduce the effect of time as a factor in the analysis. Residual diagnostics were evaluated for autocorrelation of the residuals using autocorrelation function (ACF) plots and the Breusch–Godfrey test (Hyndman and Athanasopoulos 2018), and autocorrelation was not detected. Variable selection was completed by fitting all possible models and selecting the model with the highest adjusted *R* ^2^ and predicted *R* ^2^. Generally, the model with the highest adjusted *R* ^2^ was selected, but in cases where a slight increase in adjusted *R* ^2^ was accompanied by a notable decrease in predicted *R* ^2^, the model with the highest predicted *R* ^2^ was selected. Predictors that were collinear were eliminated from the model. If two predictors were determined to be collinear, the *F* tests of significance were evaluated, and the predictor with the higher *p* value was eliminated.

### 2.6 DNA Extraction, 16S rRNA Gene Amplicon Sequencing, and Bioinformatics

Samples were selected for 16S rRNA gene sequencing based on highlighting differences between treated and control ponds. During Phase 1, samples analyzed for 16S rRNA were taken on April 9, 2020, and May 7, 2020. During Phase 2, samples analyzed for 16S rRNA were taken on May 28, 2020; June 3, 2020; July 21, 2020; and July 29, 2020. Samples taken on June 3, 2020, were from the sludge layer of the ASBs. Samples from the incubator were taken on April 9, 2020; June 7, 2020; June 28, 2020; and July 21, 2020. ASB and incubator samples were obtained in triplicate, kept in sterile 50‐mL polyethylene centrifuge tubes at 4°C, and pelletized within 24 h and stored at −20°C until DNA extraction. DNA for selected samples was extracted using a FastDNA Spin Kit for Soil (MP Biomedicals, Irvine, CA). The 16S V3 and V4 regions were PCR‐amplified with the modified 341F (5′‐CCTAYGGGRBGCASCAG) and 806R (5′‐GGACTACNNGGGTATCTAAT) universal primers for both archaea and bacteria (Yu et al. 2005). Next‐generation sequencing was performed using the Illumina MiSeq platform following the manufacturer's guidelines at MR DNA (www.mrdnalab.com, Shallowater, TX). Sequences were joined, and sequences less than 150 bp and with ambiguous base calls were removed. Then, sequences were quality filtered using a maximum expected error threshold of 1.0 and dereplicated. The dereplicated sequences were denoised; unique sequences identified with sequencing and/or PCR point errors were removed. Chimeras were then removed. Final amplicon sequence variants (ASVs) were taxonomically classified using BLASTn against a curated database derived from NCBI (www.ncbi.nlm.nih.gov). Abundances of microbial communities in each sample were exported at the genus level and visualized using the R ggplot2 package (Version 3.5.0) (Wickham 2016). Alpha and beta diversities were analyzed using the R phyloseq package (Version 1.30.0) (McMurdie and Holmes 2013). The beta diversity was illustrated in a nonmetric multidimensional scaling (NMDS) plot based on the Bray–Curtis distance, with ellipses generated assuming a multivariate t‐distribution.

## 3 Results

### 3.1 Incubator Operation

Throughout the trial period, incubator operation was monitored for temperature, DO, pH, NH_3_‐N, and filtered total phosphorus (FTP) to ensure operating conditions did not adversely affect biomass growth. The target temperature was less than 35°C, the target DO was greater than 0.5 mg/L, and the target pH was between 6 and 9. Nutrients were added as a part of the bioaugmentation product formulation. Though the product was still dosed to the wastewater treatment plant, periods when the incubator operation did not meet these targets were eliminated (41 days, from May 22 to July 3) from the dataset analyzed, to remove the effect of nonideal bioaugmentation. The biomass in the incubator was monitored using VSS, cATP, and heterotrophic plate counts (HPC) over the trial period (Table 2) and was consistent with expected biomass levels in an incubator.

**TABLE 2:** Estimates of biomass in incubator.

Parameter	Incubator measurements	Pond A measurements
VSS (mg/L)	6494 ± 5519 (13)	70.2 ± 61.4 (181)
cATP (ng/mL)	214 ± 134 (13)	91.9 ± 30.5 (98)
HPC (CFU/mL)	1.01E + 07 ± 2.17E + 06 (3)	2.33E + 05 ± 1.22E + 05 (4)

*Note:* Average ± standard deviation for combined trial data is shown. The number of measurements included is in parentheses.

The raw bioaugmentation product was 98.5%–99.5% growth media and 0.5%–1.5% by weight freeze dried *Bacillus*, according to the product safety data sheet and 16S rRNA gene sequencing of the raw bioaugmentation product (Figure S2). Although *Bacillus* and other aerobic genera were detected in the incubator, the microbial community was dominated by anaerobic bacteria (Figure S2). Aerobic DO residuals were measured consistently in the incubator. This result provides evidence that most of the active biomass in the incubator were growing attached to the growth media, which frequently sank to the bottom of the incubator where anaerobic microconditions occurred.

### 3.2 ASB Performance

#### 3.2.1 Assessment of Active Biomass

We calculated dosage ratios based on measurements indicative of biomass in the system using VSS, cATP, and HPC. Because of interference from the growth media, there was a concern that VSS was not an appropriate means of monitoring the amount of active biomass added to the ASB. cATP is considered to be directly correlated to the quantity of active biomass in the system (Whalen et al. 2006). Heterotrophic plate count (HPC) measurements were used as a secondary measure of bacteria dosed versus bacteria in the system. The mass of bacteria added to the WWTS as a percentage of the existing biomass inventory in Pond A was, on average, 0.061%, 0.0015%, and 0.028% for VSS, cATP, and HPC, respectively.

**FIGURE 2:**
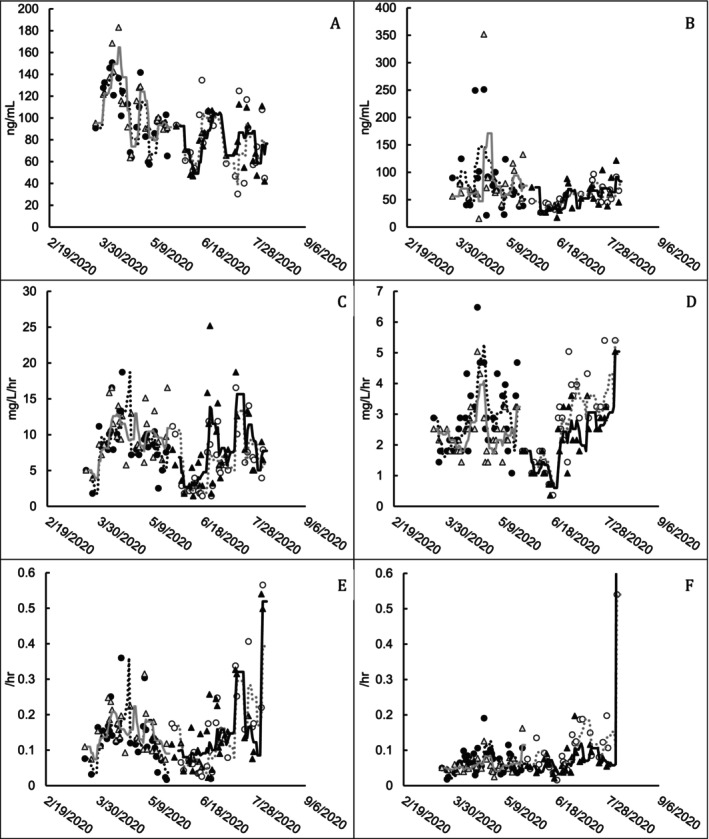
Indicator measurements of biomass activity for ponds receiving and not receiving bioaugmentation treatment. (A) Pond A cATP (ng/mL). (B) Pond B cATP (ng/mL). (C) Pond A OUR (mg/L/h). (D) Pond B OUR (mg/L/h). (E) Pond A SOUR (/h). (F) Pond B SOUR (/h). Markers represent individual data points and trend lines represent the 7‐day running average. Filled markers indicate bioaugmented pond measurements, and open markers indicate untreated pond measurements. ● Train 1 treated; △ Train 2 untreated; ○ Train 1 untreated; ▲ Train 2 treated. ⠒ Train 1 treated; gray ─ Train 2 untreated; gray ⠒ Train 1 untreated; ─ Train 2 treated.

Oxygen uptake rate (OUR) indirectly measures the activity of the biomass. cATP and SOUR (specific OUR; the OUR value normalized to VSS measurements) results for each pond and phase were not noticeably different between trains that were and were not treated with bioaugmentation (Figure 2). Statistical analysis of the effect of bioaugmentation on biomass activity is presented (Table 3) to include the final selected multiple linear model, with predictors selected, along with their *F*‐test *p* values, the adjusted *R* ^2^ of the multiple linear model, and the *p* value for the bioaugmentation treatment factor when included as a predictor in the model.

**TABLE 3:** Summary of multiple linear regression analysis for biomass activity.

Response	Predictors included in model (*F*‐test *p* values)	Adjusted *R* ^2^	*F* test for statistical significance of bioaugmentation *p* value
Pond A OUR	Train ID (0.178) Pond A ORP (0.185) Pond A conductivity (0.762) Inf. VSS (0.014)* Inf. TBOD load w/1 week lag (0.013)* Inf TSS w/1 week lag (0.011)*	0.4258	0.968
Pond A SOUR	Pond A temperature (2.21E−05)* Pond A DO (0.136) Inf. VSS (0.002)* Inf. TBOD load (0.179) Inf. VSS w/1 week lag (1.01E−05)*	0.7989	0.560
Pond A cATP	Pond A FTP (0.451) Pond A temperature (0.014)* Pond A ORP (0.056) Inf. TBOD load w/1 week lag (0.047)* Inf TSS w/1 week lag (0.319)	0.4216	0.800
Pond B OUR	Bioaug ID (0.305) Train ID (3.25E−07)* Pond B DO (0.015)* Pond B ORP (0.699) Pond B conductivity (0.003)* Pond A TBOD (7.97E−08)* Pond A VSS w/1 week lag (0.586)	0.9357	0.305
Pond B SOUR	Bioaug ID (0.003)* Train ID (0.001)* Pond B DO (0.006)* Pond B ORP (0.083) Pond A VSS (0.105) Pond A TBOD (0.559) Pond A SBOD w/1 week lag (0.138)	0.6161	0.003*
Pond B cATP	Pond B temperature (0.042)* Pond B NH3‐N (0.150) Pond B ORP (0.036)* Pond B conductivity (0.084) Pond A VSS (0.004)* Pond A SBOD (0.0002)* Pond A TBOD w/1 week lag (0.038)*	0.4212	0.398

* *p* < 0.05.

There is evidence that BOD load to each pond had a significant effect on cATP and OUR (*p* values < 0.05 in Table 3) as decreases of all activity indicators were observed during May–June 2020 when BOD load to the ASBs decreased by about 40,000 lb/d. The train sampled had a significant effect on the OUR value measured in Pond B (*p* value < 0.05 in Table 3), with Train 1 typically having a slightly higher OUR than Train 2 (Figure 2D). *F* tests for bioaugmentation effect did provide evidence that bioaugmentation had a significant lower effect on SOUR in Pond B (bioaugmentation associated with a lower SOUR of 0.04 ± 0.01/h than the control) when all other factors are controlled.

#### 3.2.2 Assessment of Long‐Term BOD Performance

**FIGURE 3:**
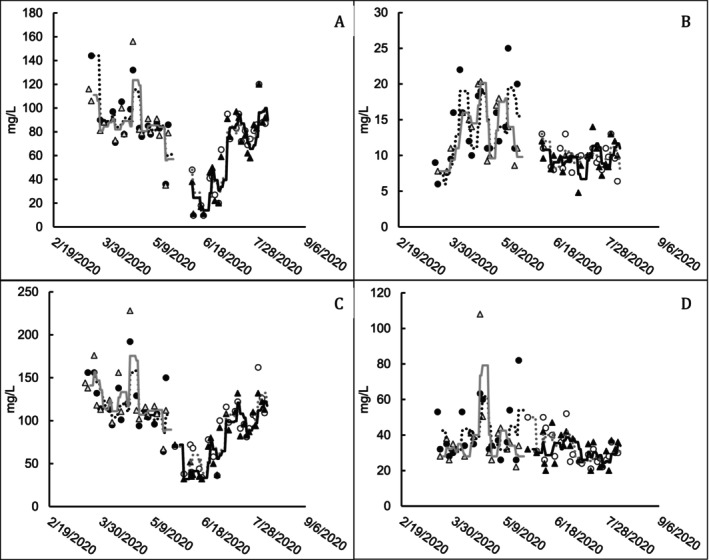
Biochemical oxygen demand measurements for ponds receiving and not receiving bioaugmentation treatment. (A) Pond A sBOD5 (mg/L). (B) Pond B sBOD5 (mg/L). (C) Pond A TBOD5 (mg/L). (D) Pond B TBOD5 (mg/L). Markers represent individual data points, and trend lines represent the 7‐day running average. Filled markers indicate bioaugmented pond measurements, and open markers indicate untreated pond measurements. ● Train 1 treated; △ Train 2 untreated; ○ Train 1 untreated; ▲ Train 2 treated. ⠒ Train 1 treated; gray ─ Train 2 untreated; gray ⠒ Train 1 untreated; ─ Train 2 treated.

Soluble BOD5 (sBOD5) represents the true efficacy of substrate removal, whereas TBOD5 represents the oxygen demand from the remaining substrate and solid particles such as biomass. The treatment facility is permitted on TBOD5. sBOD5 was nearly removed at the outlet of Pond B with greater variability at the outlet during Phase 1 (period of higher TBOD5 loading to the ponds), and Pond A outlet BOD measurements varied with influent BOD load (Figure 3).

In Ponds A and B, the influent TBOD5 load had a significant effect on outlet TBOD5 (Table S2). This is consistent with the conditions under which the trial took place. Each train received the same amount of aeration, and nutrient residuals were high enough that nutrient limitation did not occur (Mattingly 2021). *F* tests for the bioaugmentation factor indicated that bioaugmentation had a statistically significant effect on TBOD5 measurements at Pond B (*p* = 0.031). The linear model estimate of the bioaugmentation factor was 6.02 ± 2.6, indicating that when all other factors were held constant, TBOD5 was about 6 mg/L higher in the bioaugmented Pond B.

#### 3.2.3 Assessment of Long‐Term TSS Performance

**FIGURE 4:**
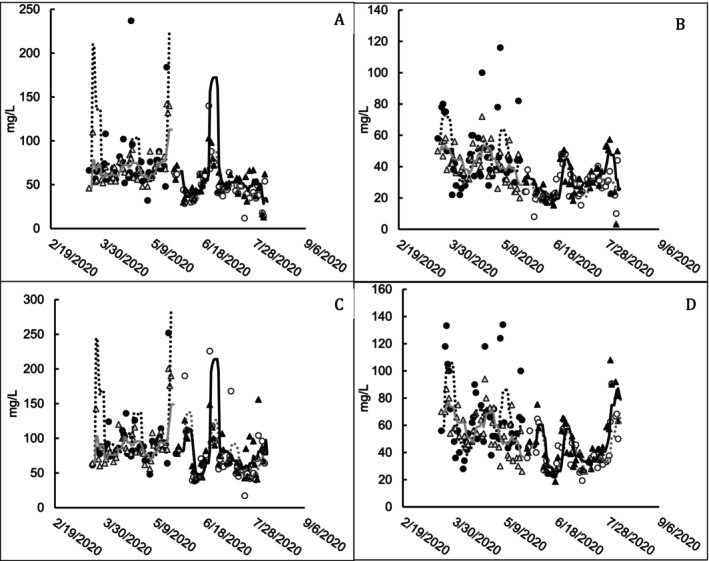
Total and volatile measurements for ponds receiving and not receiving bioaugmentation treatment. (A) Pond A VSS (mg/L). (B) Pond B VSS (mg/L). (C) Pond A TSS (mg/L). (D) Pond B TSS (mg/L). Markers represent individual data points, and trend lines represent the 7‐day running average. Filled markers indicate bioaugmented pond measurements and open markers indicate untreated pond measurements. ● Train 1 treated; △ Train 2 untreated; ○ Train 1 untreated; ▲ Train 2 treated. ⠒ Train 1 treated; gray ─ Train 2 untreated; gray ⠒ Train 1 untreated; ─ Train 2 treated. (A) Measurements above 250 mg/L VSS indicated at 250 mg/L VSS. (C) Measurements above 300 mg/L indicated at 300 mg/L VSS.

VSS represents the parameter typically used as an indicator of microbial material in a wastewater system, whereas TSS is an important permitted water quality characteristic for the treatment facility. Though the bioaugmented ponds tended to occasionally have higher VSS and TSS measurements, particularly during the period of higher BOD loading, there is no discernible difference in long‐term trends between bioaugmented and non‐bioaugmented ponds (Figure 4).

In Pond A, influent VSS (or TSS), FTP residual, and the influent TBOD load with a 1‐week lag had the most significant impact on VSS and TSS concentrations. *F* tests showed that bioaugmentation was significant at an alpha level of 0.05 for TSS in Pond B (Table S3). Bioaugmentation was associated with a higher TSS of 12.4 mg/L ± 5.2 mg/L in Pond B.

#### 3.2.4 Assessment of Long‐Term Settling Performance

**FIGURE 5:**
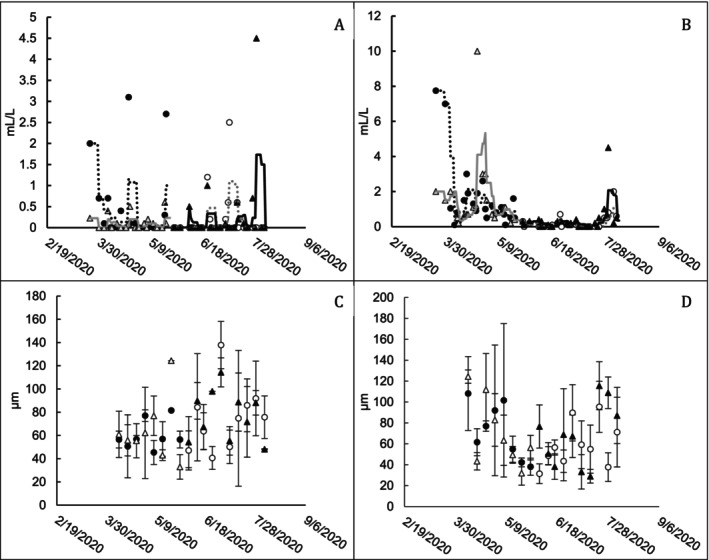
Solids settling characteristics for ponds receiving and not receiving bioaugmentation treatment. (A) Pond A settleable solids (mL/L). (B) Pond B settleable solids (mL/L). (C) Pond A D43 (μm). (D) Pond B D43 (μm). For (A) and (B), markers represent individual data points, and trend lines represent the 7‐day running average. For (C) and (D), markers represent the average of three calculations, and error bars represent the standard deviation. Filled markers indicate bioaugmented pond measurements, and open markers indicate untreated pond measurements. ● Train 1 treated; △ Train 2 untreated; ○ Train 1 untreated; ▲ Train 2 treated. ⠒ Train 1 treated; gray ─ Train 2 untreated; gray ⠒ Train 1 untreated; ─ Train 2 treated.

The settleable solids assay was used to understand if bioaugmentation affected the settling properties of solids in the mixed liquor. Any result that was reported as less than 0.1 mL/L (the lower limit of detection of the test) was assumed to be equal to 0.1 mL/L for the statistics calculations and models. The residuals of the settleable solids models showed increasing variance due to the number of values measured at the lower limit of detection. A log transformation was applied to the settleable solids numbers to meet the linear regression assumptions of homoscedasticity and normality. The D43 value as a response variable for representative floc size in microns (Figure 5C,D) shows that there is considerable variability in the estimated D43 and no clear correlation with bioaugmentation treatment. In Pond B, there were periods where the bioaugmented train appeared to have greater settleable solids than the control train. However, as BOD load decreased, this effect disappeared. In Pond A, the bioaugmented trains appeared to occasionally have higher spikes in settleable solids (Figure 5A,B). These higher peak values in settleable solids correlate with periods when the ponds had high elevated VSS and TSS. Microscopic evaluation showed that the biomass in bioaugmented and control trains was not visually different for most of the weeks measured. It was apparent that as the influent TBOD load decreased, the D43 of the particle size distribution data for Pond B decreased as well (Figure 5D). Evaluation of other indices (histograms, skewness, kurtosis, and 90th percentile equivalent diameter) did not suggest a difference between treated and untreated trains (Mattingly 2021).

Models with adequate adjusted *R* ^2^ (0.4–0.7) were not achieved for Pond A Log Settleable Solids and Pond B D43 (Table S4). In Pond A, biomass did not have adequate time to flocculate with a retention time of less than 3 days; thus, most of the solids are dispersed in nature and did not settle well. Most settleable solid values were at or close to the lower detection limit, and therefore, relationships to other predictor variables were unable to be discerned. Pond A outlet TSS and TBOD had the most significant effect on the log‐transformed settleable solids values measured in Pond B. Influent solids (either VSS or TSS) appeared to have the greatest correlation on the D43 value, particularly for Pond B. In Pond B, the influent VSS is most affected by the TBOD load to the WWTP. At higher TBOD loads, Pond A VSS and Pond B D43 are higher. The D43 data are consistent with microscopic analysis that found the majority of flocs in both ponds to be less than 150 μm. Bioaugmentation was not significant at an alpha level of 0.05.

#### 3.2.5 Assessment of Whole Effluent Toxicity

For the *Ceriodaphnia dubia* bioassays, the concentration of Pond B outlet wastewater that caused a 25% decrease in reproduction was calculated (IC25) and compared for bioaugmented and control trains (Table 4).

**TABLE 4:** Summary of WET IC25 (%).

Sample	Bioaugmented train (upper and lower 95% CI)	Control train (upper and lower 95% CI)
3/30/2020	73.5 (69.3, 77.6)*	91.0 (87.7, 94.3)
4/13/2020	N/A^a^	N/A
5/1/2020	4.6 (0.2, 8.9)*	71.3 (69.2, 73.4)
5/10/2020	70.9 (62.7, 79.2)	73.4 (68.4, 78.3)
6/19/2020	65.9 (61.0, 70.8)	58.9 (54.7, 63.0)
7/11/2020	59.6 (56.9, 62.2)*	87.1 (83.7, 90.5)
7/28/2020	N/A	N/A

^a^ N/A means that there was a less than 25% decrease in reproduction from the control to the 100% wastewater test.

* Significant difference, paired *t* test, *p* < 0.05.

Three of the seven comparative bioassays showed a significant difference between the bioaugmented and control trains. Where differences between the bioaugmented and control trains occurred, bioaugmentation appeared to be associated with a lower IC25. In four of the seven comparative bioassays, there was no significant difference between the bioaugmented and control trains.

### 3.3 Assessment of Microbial Community Structure in the Incubator and ASBs

**FIGURE 6:**
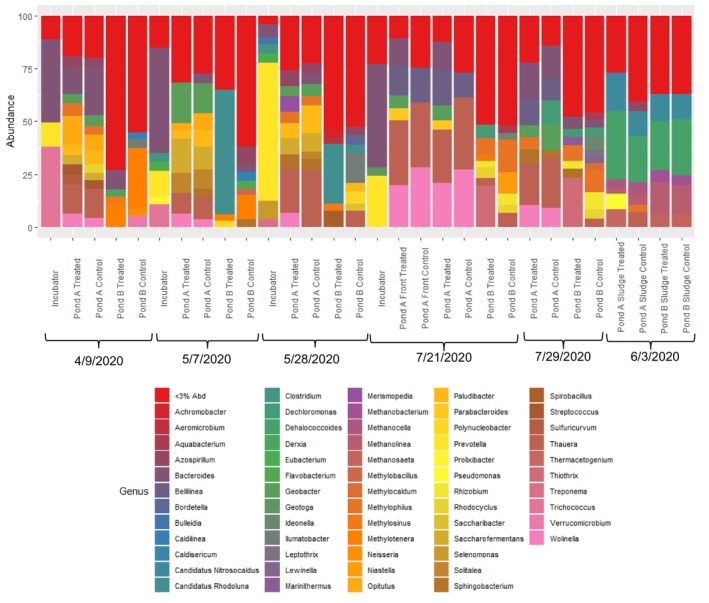
Relative abundances of dominant bacterial genera (defined as greater than 3% relative abundance).

Plots of relative abundance (defined as the number of sequence reads for a genera divided by the total number of sequence reads in a sample) for each of the sampling dates (Figure 6) present the most abundant genera, with different bars of the same color representing different species of the same genera.

*Thauera* was dominant in Pond A in four out of five samples. *Thauera* is a facultative aerobic heterotroph, capable of using oxygen, nitrate, or nitrite as an electron acceptor (Thomsen et al. 2007). *Bacteroides* was dominant in Pond A in samples collected April 9, 2020; July 21, 2020; and July 29, 2020. In the samples collected July 21, 2020, *Bacteroides* was the most abundant genus in the bioaugmentation incubator and accounted for approximately 13% of the relative abundance of bacteria in the bioaugmented train but only accounted for about 1% of the relative abundance in the control train. *Bacteroides* genera are anaerobic fermenters (Song et al. 2015) and are often present in methanogenic reactors (Ueki et al. 2008). *Geobacter* was dominant in Pond A on June 7, 2020, and July 29, 2020. *Geobacter* is strictly anaerobic with Fe (III) as the electron acceptor (Coates and Lovely 2015) and capable of nitrogen fixation (Ueki and Lovley 2010). *Wolinella* and *Bellilinea* were dominant in Pond A on July 21, 2020, and July 29, 2020. *Wolinella* is anaerobic and capable of using hydrogen gas or formate as an electron donor, with fumarate or nitrate being used as the electron acceptor (Tanner and Paster 1992). *Bellilinea* is also strictly anaerobic, as well as neutrophilic and thermophilic, and forms filaments (Nierychlo et al. 2020). Despite the aeration provided in Pond A, the microbial community was dominated by anaerobic bacteria. This suggests that anaerobic conditions in the settled solids layer at the bottom of Pond A likely determined the microbial community composition in Pond A.

*Methylotenera* was dominant in Pond B on April 9, 2020, and June 7, 2020. *Methylotenera* is aerobic and uses methylamine (potentially also methanol) as an electron donor and carbon source (Nierychlo et al. 2020). On June 7, 2020, and June 28, 2020, *Candidatus Rhodoluna* was dominant in Pond B. On June 28, 2020, *Candidatus Rhodoluna* was dominant in the treated train Pond B. This genus has previously been identified in freshwater systems and is aerobic (Taipale et al. 2016). *Ilumatobacter* was also dominant in Pond B of the control train on June 28, 2020, and is aerobic, previously isolated from seawater (Nierychlo et al. 2020). *Methylophilus* was dominant in Pond B on July 21, 2020, and July 28, 2020. This genus is aerobic and utilizes methanol as its only source of carbon and energy (Jenkins et al. 1987). *Thiothrix* was dominant in the Pond B treated train on July 21, 2020, and July 29, 2020. *Thiothrix* is a filamentous organism capable of oxidizing reduced sulfur compounds and is generally aerobic, although capable of using nitrate as an electron acceptor under anaerobic conditions (Nierychlo et al. 2020). Pond B were dominated by aerobic bacteria. During the week of July 29, 2020, the treated Pond B had higher effluent VSS and TSS than the control pond. The high abundance of *Thiothrix* in the treated pond may indicate that the higher VSS and TSS in the bioaugmented pond are from filamentous growth rather than an effect of the bioaugmentation (*Thiothrix* was not present in the bioaugmentation incubator). Although *Bacillus* was present in the lagoons, it was not part of the dominant genera.

**FIGURE 7:**
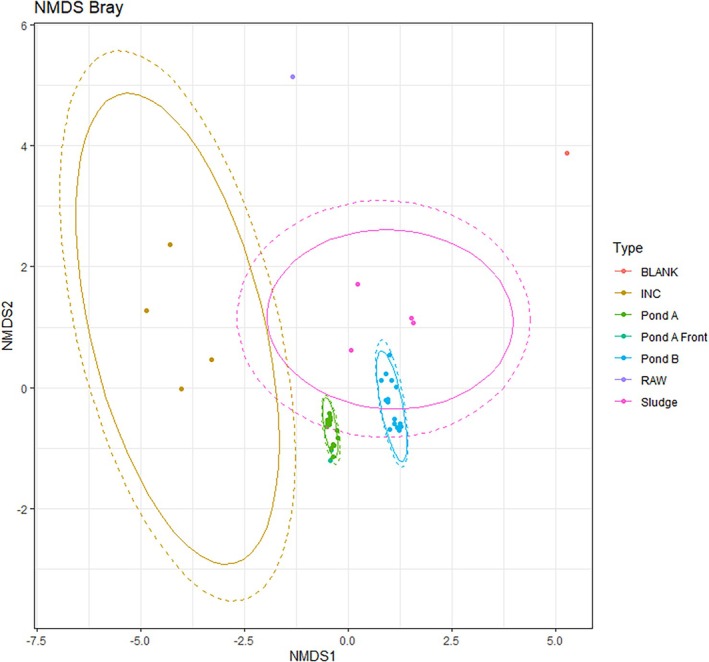
Nonmetric multidimensional scaling with Bray–Curtis dissimilarity. Ellipses represent the 95% confidence interval around centroids.

Beta diversity was explored through nonmetric multidimensional scaling (NMDS) using the Bray–Curtis dissimilarity (Figure 7), with ellipses representing the 95% confidence intervals around their centroids. No significant differences were found between treated and control train samples. However, samples from Pond A, Pond B, and the incubator can be clearly distinguished.

## 4 Discussion

Despite widespread use across the industry, there exist few demonstrations of bioaugmentation, where results were analyzed independently by a third party or published in the peer‐reviewed literature (Table 5).

**TABLE 5:** Literature summary of bioaugmentation in pulp and paper industry wastewater treatment.

Source	Bioaugmentation target	WWTS	Study type	Dosage/biomass inventory ratio (% solids mass basis, unless otherwise specified)	Major results
Sonkar et al. (2020)	General	—	Bench Scale (Batch)	8% (volume basis)	Bioaugmentation improved performance (compares batch tests to full scale final effluent)
Bailón‐Salas et al. (2017)	General	ASB	Full Scale	Not reported	PCR‐DGGE analysis showed that the dominant bacteria in the lagoons were not similar to the commercial inoculum tested. The commercial inoculum bacteria were found in the lagoons. Effect on treatment efficiency not evaluated.
Chen et al. (2012)	Lignin	SBR	Bench Scale	~0.000004%	Bioaugmentation improved lignin, COD and BOD removal. However, it also caused higher SVI and bulking.
Yu and Mohn (2002)	Resin acid	Aerated lagoon	Bench scale (batch)	Not reported	Bioaugmentation improved resin acid removal, but not TOC. Bioaugmentation of resin acid degrading bacterial may be most useful following a pH stress event.
Yu and Mohn (2001)	Resin acid	SBR	Bench scale	Not reported	Bioaugmentation improved resin acid removal, but not TOC. The bioaugmented strains were not present in the microbial community before bioaugmentation but were after.
van Ginkel et al. (1999)	EDTA	AST	Bench scale	N/A Investigated bioaugmentation organism only (not when applied to a native mixed community)	Bioaugmentation resulted in EDTA removal (not controlled).
Conners and Foster (1998)	General	Aerated lagoon	Bench	Not reported	Tests of Bioaugmentation Product Incubator, not of effect on WWTS
Whiteman et al. (1998)	General	Aerated lagoon	Bench (batch)	N/A Investigated bioaugmentation organism only (not when applied to a native mixed community)	Native microbial population was more effective than 5 bioaugmentation product formulations on the market.
Buckley (1992)	N/A	N/A	N/A	N/A	Review of Experience to Date
Whiteman and Holzer (1992)	General	ASB	Full scale	Not reported	Effluent BOD decreased by 43% after introduction of Bioaugmentation Product. No evaluation of influent loading.
Christiansen et al. (1992)	General	ASB	Full scale	Not reported	BOD removal increased from 76% to 78% (no statistical test completed on data), ATP monitoring suggested higher levels of microorganisms after bioaugmentation.
Blake and Zuncich (1991)	AOX	ASB	Bench (full scale trial started but no results presented)	Not reported	No control, but bioaugmented batch tests suggested that a mixture of bioaugmentation products had better AOX removal than just one of the products alone.
NCASI Technical Bulletin No. 438, 1984 (Buckley 1984)	General	AST	Full scale	0.03%–0.1%	Bioaugmentation had no effect on BOD. A statistically significant effect on TSS was found, but further monitoring of the system suggested that this effect may have been due to differences in trains rather than bioaugmentation.
This study	General	ASB	Full scale	0.002%–0.06%	Bioaugmentation had no practically significant effect on BOD or TSS.

Abbreviations: ASB, aerated stabilization basin; AST, activated sludge, SBR, sequencing batch reactor.

Pulp and paper wastewater studies at full scale are scarce, have only evaluated general bioaugmentation, and often are complicated by poor controls (controls in time with inadequate characterization before and after) or lack of statistical analysis. For example, in a study that potentially demonstrated a significant benefit of bioaugmentation (Whiteman and Holzer 1992), influent BOD load to the system was not monitored, and it is not known whether this should have been a factor accounted for in analysis. Bailón‐Salas et al. (2017) used PCR‐DGGE to study the microbial community structure of two lagoons in series at a mill in Mexico, finding that the microbial community in the lagoons contained bioaugmented bacteria, but none of the dominant genera matched the bioaugmented strains. The effect of bioaugmentation on treatment performance was not reported. It is also doubtful that the amounts of bioaugmented cells are typically high enough to change the microbial community or performance of the system. For example, in a full‐scale study of bioaugmentation of a pulp and paper mill activated sludge plant, the bioaugmentation dose ranged from 1 to 3 mg/L, with an initial dose of 11 mg/L (Buckley 1984). The design MLSS was 3000 mg/L, meaning that the percentage of bacteria added to the system compared to the biomass in the system ranged from 0.03% to 0.1%, with a 1‐day inoculation of 0.0037‐mg bioaugmentation biomass/mg activated sludge biomass.

Few of the pulp and paper wastewater studies in the literature have documented dosage in relation to existing biomass inventory, and the methods of doing so vary (i.e., plate counts, VSS, and molecular methods). None of the studies specifically evaluated the required dosage to either improve treatment performance with respect to an effluent target or impart a change to the structure of the microbial community. At the bench scale level, the microbial community is not subjected to the multitude of temporal and environmental variations that it is subjected to at the full‐scale level (Madsen 1998). Thus, the required sustained dosage of a bioaugmentation product to impart a lasting change in the microbial community may be higher at the full‐scale level. A qualitative comparative analysis identified factors that led to a successful bioaugmentation application as the use of a targeted approach, the use of one bioaugmentation strain (as opposed to a consortia), and the practice of culturing the bioaugmentation product with the treatment target prior to application (Mattingly 2021). Of the published studies in the pulp and paper industry, many, including this one, did not include a targeted approach (meaning a specific chemical rather than generalized BOD and TSS performance). In our study, the addition of the generalized bioaugmentation product did not improve performance of the ASBs at dosages consistent with what is typically employed (see Section 2). The mass of bacteria added to the aeration basin from the incubator ranged from 0.002% to 0.06% of the aeration basin mass, depending on biomass measurement method. We did not evaluate higher dosage ratios as the goal of this study was to assess the efficacy of current industry practices. A common reason for failure of a bioaugmentation product is limited growth of the product in the treatment plant. The environmental conditions and microbial community analysis of the first set of ponds showed that these ponds favor anoxic and anaerobic bacteria that tolerate moderate to high temperatures. Though the onsite incubator was employed to acclimate the bioaugmented bacteria to the environmental conditions onsite, the bioaugmentation product designed for aerobic conditions may have been uncompetitive with microorganisms better suited to the environmental conditions of the ponds. Pond A often had low dissolved oxygen concentrations and were dominated by anaerobic bacteria. Some of the dominant genera in the incubator were also dominant in Pond A.

Active biomass concentrations, quantified by cATP, were not associated with the bioaugmentation factor in the multiple linear regression models for either Pond A or B. The statistical estimate of bioaugmentation effects suggested that bioaugmentation to a train correlated with lower Pond B SOUR, higher Pond B TBOD5, and higher Pond B TSS. Because no significant effect was found for sBOD5, it can be inferred that the higher TBOD5 is due to particulate BOD5. Analysis of microbial community data showed that although some of the dominant genera in the incubator were detected in the ponds, bioaugmentation did not cause any large changes in the microbial community structure in the ponds. In addition, results from the microbial community analysis suggest that the growth of *Thiothrix* filamentous bacteria likely contributed to the difference in pond performance observed in Phase 2. *Thiothrix* is a common filament present in pulp and paper industry wastewaters (Jenkins et al. 2004), owed to the tendency of these wastewaters to turn septic (high sulfate and readily biodegradable BOD concentrations). The conditions for this were demonstrated during this study. Low dissolved oxygen concentrations were observed in Pond A, leading to sulfate reduction and the consequential bloom of *Thiothrix* in the subsequent aerobic pond. It is curious that the *Thiothrix* bloom occurred only in the bioaugmented pond, and this phenomenon requires further investigation. It may have been related to the bioaugmentation product itself but may have also been related to differences between the two trains. Tracer studies of the two trains showed near identical hydraulic characteristics but did estimate a slightly higher mean residence time for Pond A in Train 2 (the bioaugmented one during the *Thiothrix* bloom). The added time in Pond A, combined with warmer summer temperatures, may have allowed for greater anaerobic activity, resulting in the right conditions for *Thiothrix* proliferation in Pond B.

Overall, we attribute the failure of the bioaugmentation product to impact the wastewater treatment process to the small dosage amount when compared to the native biomass inventory as well as the generalized nature of the application. Bioaugmentation, although not successful in this instance, may be successful with a sufficient dosage and when applied for a more targeted purpose. Targeted applications where bioaugmentation may be successful could include during start‐up of a wastewater treatment process or when degradation of a specific compound is required that the naturalized community is not addressing (i.e., ammonia, resin acids, and AOX). Future studies should address required dosages to observe an impact in the removal of a target compound and generate a change in the structure of the microbial community. For facilities considering implementation of a bioaugmentation product, we recommend the following steps:

- Consider if there are volume, oxygen or nutrient limitations that require corrections before implementing generalized bioaugmentation to improve BOD and TSS removal.
- Consider use of bioaugmentation for targeted applications rather than for long‐term generalized applications.
- Estimate the ratio of dosed bacteria to the biomass inventory.
- Request bioaugmentation vendors provide evidence that the proposed dosage will result in a change in the microbial community function and the basis for the calculation of the proposed dosage.
- Conduct sufficient measurements prior to and during bioaugmentation implementation to statistically evaluate an impact in targeted final effluent characteristics.

Future studies should focus on determining a biomass/inventory ratio that imparts a change to the microbial community and evaluating the relationship of simpler bench‐scale experiments in determining dosage ratios to results at full scale.

## Author Contributions

**Amanda Johansen Mattingly:** conceptualization, data curation, formal analysis, investigation, methodology, funding acquisition, resources, validation, visualization, writing – original draft, writing – review and editing. **Philip Pagoria:** conceptualization, methodology, funding acquisition, resources, supervision, writing – review and editing. **James Palumbo:** conceptualization, methodology, funding acquisition, resources, writing – review and editing. **Francis L. de los Reyes III:** conceptualization, methodology, project administration, supervision, funding acquisition, resources, validation, writing – review and editing.

## Conflicts of Interest

The authors declare no conflicts of interest.

## Supporting information


**Table S1:** Survey of bioaugmentation dosing at pulp and paper mill WWTPs.
**Table S2:** Summary of multiple linear regression analysis for BOD5.
**Table S3:** Summary of multiple linear regression analysis for TSS and VSS.
**Table S4:** Summary of multiple linear regression analysis for D43 and settleable solids.
**Figure S1:** Bioaugmentation system process flow diagram.
**Figure S2:** Bar plot of Genus identifications in incubator (Abundance is fraction of ASVs).
**Figure S3:** Ponds A alpha diversity.
**Figure S4:** Ponds B alpha diversity.

## Data Availability

The data that support the findings of this study are available on request from the corresponding author. The data are not publicly available due to privacy or ethical restrictions.
