# Supplementary material for: Does Continuous Bioaugmentation of Aerated Stabilization Basins Improve Performance? A Field Scale Trial With a Control
Source: Water Environ Res. 2025 Nov 12;97(11):e70202. doi: 10.1002/wer.70202 (PMC12606700; doi:10.1002/wer.70202)
Supplement: Supplementary file 1 — Table S1: Survey of bioaugmentation dosing at pulp and paper mill WWTPs. Table S2: Summary of multiple linear regression analysis for BOD5. Table S3: Summary of multiple linear regression analysis for TSS and VSS. Table S4: Summary of multiple linear regression analysis for D43 and settleable solids. Figure S1: Bioaugmentation system process flow diagram. Figure S2: Bar plot of Genus identifications in incubator (Abundance is fraction of ASVs). Figure S3: Ponds A alpha diversity. Figure S4: Ponds B alpha diversity. [file WER-97-e70202-s001.docx]

**Supplemental Information**

**Does continuous bioaugmentation of aerated stabilization basins improve performance? A field scale trial with a control**

Amanda Johansen Mattingly^ab^, Philip Pagoria^b^, James Palumbo^b^, and Francis L. de los Reyes III*^a^

^a^Department of Civil, Construction, and Environmental Engineering, North Carolina State University, 915 Partners Way, Raleigh, NC 27695-7908

^b^National Council for Air and Stream Improvement, Inc., 1513 Walnut Street, Suite 200, Cary, NC 27511-5927

**Table S1. Survey of bioaugmentation dosing at pulp and paper mill WWTPs**

| **Wastewater Treatment Plant** | **Bioaugmentation Dose** | **Bioaugmentation Dose/Avg. Inf. BOD** |
| --- | --- | --- |
| A | 0.9 kg/d | 0.2 kg/10,000 kg |
| B | 4.5 kg/d | 1.2 kg/10,000 kg |
| C | 3.6 kg/d | 0.6 kg/10,000 kg |
| D | 11.3 kg/d | 2.8 kg/10,000 kg |
| E | 5.2 kg/d | 1.0 kg/10,000 kg |
| F | 3.6 kg/d | 0.9 kg/10,000 kg |
| G | 22.7 kg/d | 4.3 kg/10,000 kg |

**Table S2. Summary of multiple linear regression analysis for BOD5**

| **Response** | **Predictors Included in Model (F-test p-values)** | **Adjusted R^2^** | **F-Test for Statistical Significance of Bioaugmentation p-value** |
| --- | --- | --- | --- |
| Pond A TBOD5 | Pond A Temperature (0.038)*  Bioaug. ID (0.213)  Inf. TBOD Load (0.007)*  Pond A ORP (0.083)  Pond A Conductivity (0.214)  Inf. TBOD Load w/ 1 Week Lag (0.207) | 0.7591 | 0.213 |
| Pond A sBOD5 | Inf. TBOD Load (0.077)  Pond A ORP (0.214)  Pond A Conductivity (0.218) | 0.4973 | 0.840 |
| Pond B TBOD5 | Bioaug. ID (0.031)*  Pond A TBOD (4.43E-07)*  Pond B NH3-N (0.001)*  Pond B Conductivity (0.162) | 0.7748 | 0.031* |
| Pond B sBOD5 | Pond B Temperature (0.241)  Pond A SBOD (0.184)  Pond B NH3-N (0.013)*  Pond B DO (0.235)  Pond A TBOD w/ 1 Week Lag (0.369) | 0.6149 | 0.596 |

*Significant factor at alpha = 0.05

**Table S3. Summary of multiple linear regression analysis for TSS and VSS**

| **Response** | **Predictors Included in Model (F-test p-values)** | **Adjusted R^2^** | **F-Test for Statistical Significance of Bioaugmentation p-value** |
| --- | --- | --- | --- |
| Pond A TSS | Pond A NH3-N (0.206)  Pond A FTP (0.007)*  Pond A DO (0.107)  Inf. TSS (5.09E-06)*  Inf. TSS w/ 1 Week Lag (0.029)*  Inf. TBOD Load w/ 1 Week Lag (0.307) | 0.842 | 0.429 |
| Pond A VSS | Pond A NH3-N (0.011)*  Pond A FTP (5.18E-09)*  Inf. VSS (5.42E-05)*  Inf. VSS w/ 1 Week Lag (0.069)  Inf. TBOD Load w/ 1 Week Lag (4.80E-05)* | 0.8836 | 0.283 |
| Pond B TSS | Bioaug. ID (0.029)*  Pond B ORP (0.211)  Pond B NH3-N (0.046)*  Pond A TBOD (0.010)* | 0.4542 | 0.029* |
| Pond B VSS | Bioaug. ID (0.115)  Pond B ORP (0.081)  Pond A VSS w/ 1 Week Lag (0.035)*  Pond A TBOD (0.013)* | 0.4871 | 0.115 |

*Significant factor at alpha = 0.05

**Table S4. Summary of multiple linear regression analysis for for D43 and settleable solids**

| **Response** | **Predictors Included in Model (F-test p-values)** | **Adjusted R^2^** | **F-Test for Statistical Significance of Bioaugmentation p-value** |
| --- | --- | --- | --- |
| Log(Pond A Settleable Solids) | Inf. TBOD Load w/ 1 Week Lag (0.204)  Pond A ORP (0.027)* | 0.1943 | 0.350 |
| Pond A D43 | Pond A Temperature (0.014)*  Inf. TSS w/ 1 Week Lag (0.024)* | 0.4566 | 0.493 |
| Log(Pond B Settleable Solids) | Pond B Temperature (0.273)  Bioaug. ID (0.056)  Pond A TSS (0.028)*  Pond A TSS w/ 1 Week Lag (0.024)*  Pond A TBOD (0.002)*  Pond A TBOD w/ 1 Week Lag (0.003)* | 0.6355 | 0.056 |
| Pond B D43 | Pond A VSS (0.002)*  Pond A VSS w/ 1 Week Lag (0.005)* | 0.322 | 0.953 |

*Significant factor at alpha = 0.05


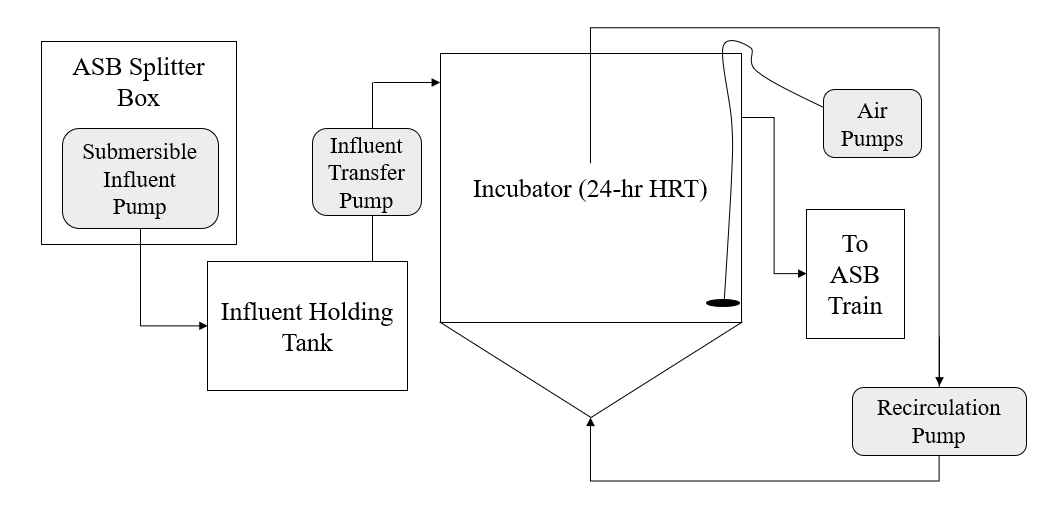


Figure S1. Bioaugmentation system process flow diagram


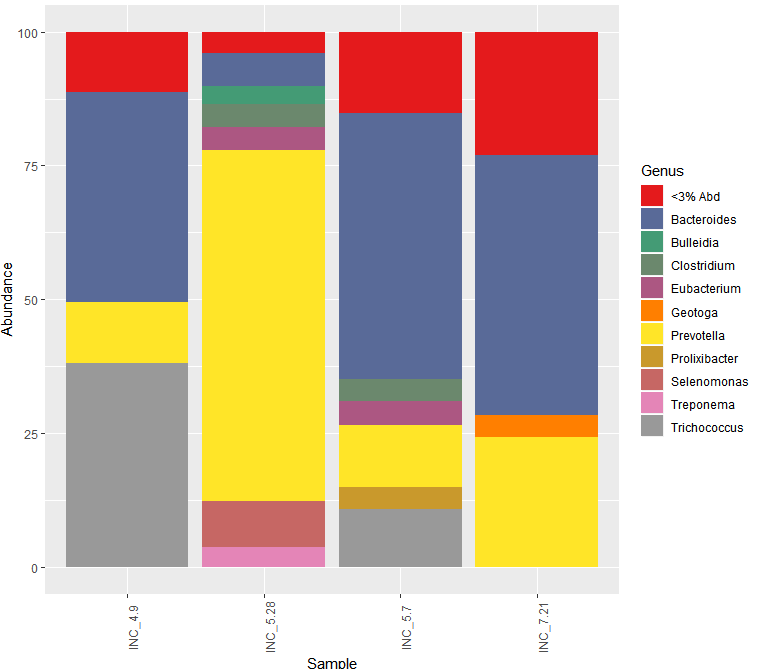


Figure S2. Bar plot of Genus identifications in incubator (Abundance is fraction of ASVs)

Alpha Diversity and Functional Organization: Alpha diversity was analyzed using the P phyloseq package (version 1.30.0) (McMurdie and Holmes, 2013). Alpha diversity is compared for samples where bioaugmentation was expected to have the greatest effect (time points after bioaugmentation was employed for at least 1 month) in the following figures.


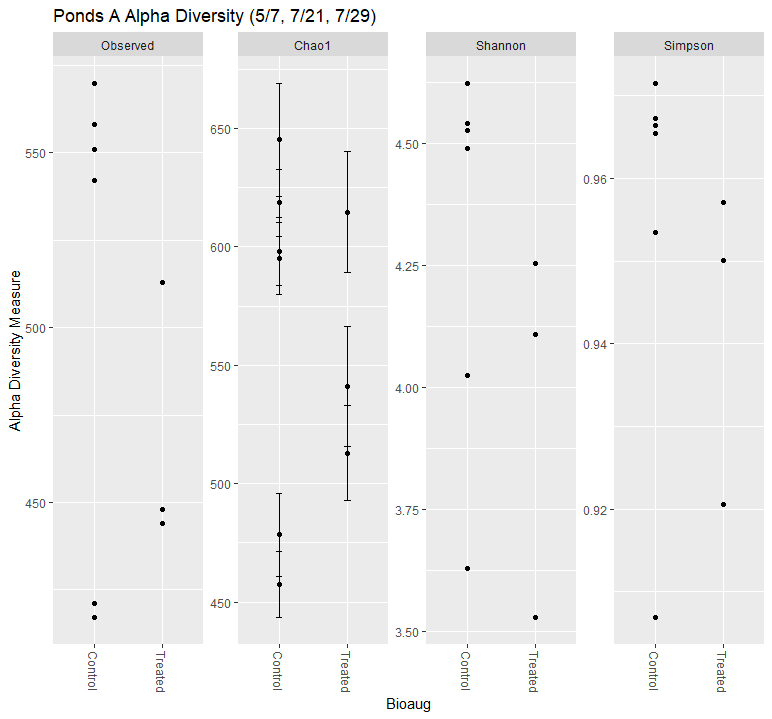


Figure S3. Ponds A alpha diversity


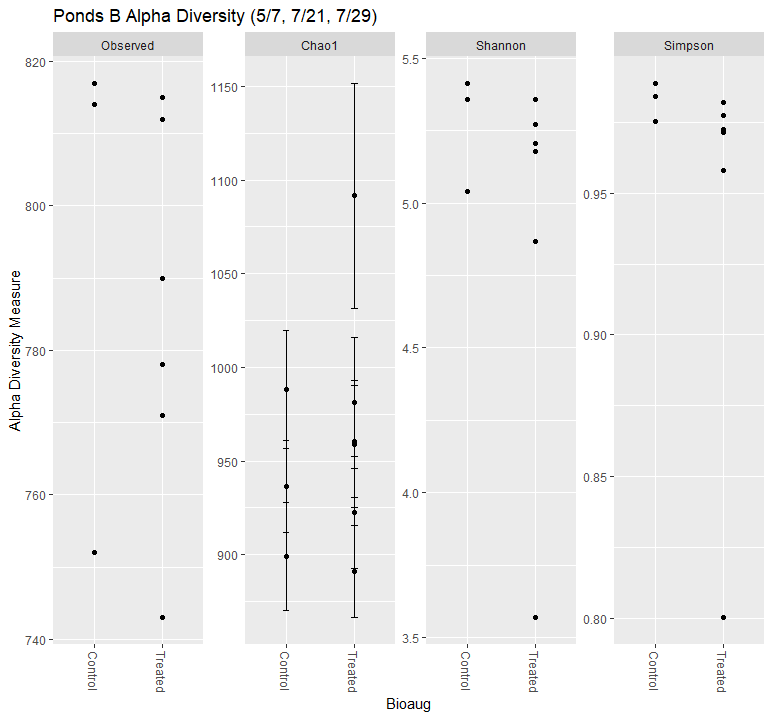


Figure S4. Ponds B alpha diversity

References:

McMurdie and Holmes, “phyloseq: An R Package for Reproducible Interative Analysis and Graphics of Microbiome Census Data.” PLoS ONE, 2013.
